# Evaluation of the Chemosensoric Properties of Commercially Available Dog Foods Using Electronic Sensors and GC-MS/O Analysis

**DOI:** 10.3390/molecules28145509

**Published:** 2023-07-19

**Authors:** Hyangyeon Jeong, Moon Yeon Youn, Sojeong Yoon, Seong Jun Hong, Seong Min Jo, Kyeong Soo Kim, Eun Ju Jeong, Hyun-Wook Kim, Eui-Cheol Shin

**Affiliations:** 1 Department of GreenBio Science, Gyeongsang National University, Jinju 52725, Republic of Korea; 2 Agri-Food Bio Convergence Institute, Gyeongsang National University, Jinju 52725, Republic of Korea; 3 Department of Pharmaceutical Engineering, Gyeongsang National University, Jinju 52725, Republic of Korea; 4 Department of Plant & Biomaterials Science, Gyeongsang National University, Jinju 52725, Republic of Korea; 5 Department of Animal Science & Biotechnology, Gyeongsang National University, Jinju 52725, Republic of Korea; 6 Division of Food Science and Technology, Gyeongsang National University, Jinju 52725, Republic of Korea

**Keywords:** dog foods, E-tongue, E-nose, GC-MS, GC-O

## Abstract

Pet owners think of their animals as part of their family, which further promotes the growth of the pet food market, encouraging pet owners to select nutritious, palatable, and high-quality foods for pets. Therefore, the evaluation of taste and volatile compounds in pet foods is essential to improve palatability. In this study, the sensory characteristics of taste and odor compounds in 10 commercially available dry dog foods were investigated using electronic tongue (E-tongue), electronic nose (E-nose), gas chromatography–mass spectrometry (GC-MS), and gas chromatography–olfactometry (GC-O). Dry dog foods were separated based on the sensory properties of taste and volatile compounds through the multivariate analysis of integrated results of the E-tongue and E-nose. A total of 67 odor active compounds were detected through GC-MS and GC-O, and octanal, nonanal, 2-pentyl furan, heptanal, and benzaldehyde were identified as key odor compounds which may have positive effects on food intake. The multivariate analysis was used to classify samples based on key odor compounds. Volatile compounds responsible for aroma properties of samples were evaluated using GC-O and multivariate analysis in this present study for the first time. These results are expected to provide fundamental data for sensory evaluation in producing new dog foods with improved palatability.

## 1. Introduction

With the growing popularity of companion animals around the world, the pet food market is experiencing significant growth and has great potential for sustained growth. According to the American Pet Products Association(APPA), the pet industry reached the highest sales of USD 123.6 billion in 2021, of which food and treats were USD 50 billion in the USA [1]. Among many types of pets, dogs are the most popular pets in the world, with 38.4 percent of respondents owning them, followed by 25.4 percent of cats [2]. Many pet owners consider their animals as part of the family, which further promotes the growth of the pet food market, encouraging pet owners to choose nutritious and high-quality foods for their pets [3,4].

Commercially available pet foods vary according to the ingredients included and the processing methods used. Pet food can be classified into three types: moist, semi-moist, and dry according to the final moisture content [5,6,7,8]. Moist pet foods with a moisture content of 65% or more generally contain high levels of proteinaceous ingredients such as meat, fish, or meat by-products and have a limited shelf-life after opening. Semi-moist foods have a final moisture content between 20% and 65% and contain a high content of simple carbohydrates which makes them more palatable [7,8]. Dry pet foods contain less than 20% moisture and are usually produced in the form of pellets and kibbles that are baked or extruded [7]. Dry dog foods have a long shelf-life and microbiological stability due to their low levels of water content [9]. Dry form is the most popularly used and constitutes the major segment of the pet food market since it is convenient to store and feed. However, dry pet foods are least palatable among the three types [7].

Palatability describes with how easily a food is accepted, and the overall palatability of dog food is influenced by its appearance, smell, taste, and texture of the product [10]. Dog foods are produced by blending various ingredients including meat, meat by-products, fish, vegetables, cereals, vitamins, and minerals to meet the right balance of nutrients and good palatability. Factors of dog food formulation such as quality and quantity of ingredients as well as the condition of manufacturing process contribute to the properties and the palatability of the final product [11,12]. The combination of olfaction and taste creates a flavor, which plays a critical role in the sensory perception and the consumption of a food [13,14]. It has been reported that olfactory capability of dogs is far more developed than that of humans, while the sense of taste has more advanced in humans compared to dogs [15,16]. Therefore, sensory perception may vary according to species and olfactory sense appears to play an important role in the sensory experience of food selection and intake in the case of dogs [17,18]. In a study by Houpt et al., olfaction was found to be critical to discriminate between foods with different meat sources [19]. Jackson et al. reported that dogs were capable of using olfactory cues to distinguish between different quantities of food relying on the type of food served and the odors of food emitted [20]. Additionally, the combination of food preference and olfactory discrimination tests demonstrated that first choice ranking and food intake ratio ranking were strongly linked, suggesting that odor perception affected the dogs’ food choice and food consumption [21]. Thus, it is necessary to evaluate volatile compounds of dry dog foods to find a way to improve overall palatability by dogs.

In the food industry, analytical techniques have been developed to characterize the flavor of food products, and recently E-tongue, E-nose, and GC-MS were widely applied to analyze the sensory properties and the quality of foods [22,23,24]. E-tongue and E-nose consist of array of sensor associated with a pattern recognition system used to detect and distinguish taste and odor substances of food matrices and are utilized to classify the food samples including vegetables, fruits, fish, meats, and beverages [25,26]. The basic principle of E-tongue is to measure and quantify the sensitivity between the component representing the taste of the sample and the electronic sensor, so that data with objectivity and reproducibility can be obtained through E-tongue analysis [27]. Using sensors related to volatile compounds, the E-nose analyzes individual volatile compounds present in food in a short period of time and simultaneously detects patterns of overall scent components to provide results [27]. The best way to evaluate the effect of taste and odor in food on dogs’ food intake and selection is to conduct a preference test or acceptance test in dogs. However, animal palatability testing is challenging because of controlling individual variability among dogs and is additionally time-consuming and expensive [10]. Thus, electronic sensors such as E-tongue and E-nose have been used to analyze the taste and aroma components of dog food in a faster and more objective manner [26].

GC-MS is commonly used to identify different substances within a liquid or volatile sample and headspace–solid–phase microextraction is a technique used to extract and isolate volatile compounds in a rapid and simple way [22,28,29]. E-tongue, E-nose, and HS-SPME combined with GC-MS have been applied to characterize the taste and volatile compounds of pet foods [30,31,32,33]. The combination of an E-nose and an E-tongue was used to discriminate canned pet foods with different composition and used to assess the flavor of pet food during the food development steps [30,31]. In addition, recent studies investigated odor active compounds in dry dog food using HS-SPME coupled with GC-MS [32,33]. In a study by Yin et al., volatile compounds correlated with the intake ratio of dog food were identified, and the contribution of key volatile compounds on the palatability of dog food were verified by the preference test [33]. Although GC–MS is an effective analytical technique for identifying volatile compounds in complex mixtures, it is unable to determine properties of the odor compounds. GC-O has been employed for identifying odor active compounds from odorless volatiles in food products [34]. GC-O is a powerful analytical tool which uses human nose as a sensitive and specific detector, and it has been widely used to detect the volatile compounds in food samples [28]. To the best of our knowledge, there are no studies reported on odor evaluation of commercial dry dog foods using GC-O techniques.

In this context, the objectives of this study were to: (1) characterize the taste pattern and the volatile compounds in commercially available dry dog foods using E-sensors and GC-MS; (2) examine which volatile compounds have influence on the olfactory profiles of dog foods through GC-O analysis using human senses; and (3) evaluate the pattern and dissimilarity among dog food samples according to the sensory characteristics of taste and volatile compounds through the multivariate analysis of chemical sensory analysis data.

## 2. Results and Discussion

### 2.1. E-Tongue Analysis

**Figure 1:**
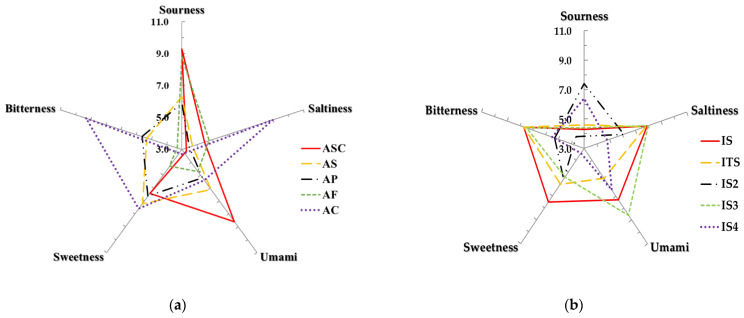
Taste components in dog food by E-tongue. (**a**) Asian dog food. (**b**) International dog food. ASC: Asian_salmon and chicken, AS: Asian_salmon, AP: Asian_poultry, AF: Asian_fish, AC: Asian_chicken, IS: International_salmon, ITS: International_turkey and salmon, IS2: International_salmon, IS3: International_salmon, IS4: International_salmon.

The E-tongue analysis system measures and quantifies the sensitivity between the component representing the taste of the sample and the electronic sensor. All terms in the results are abbreviated for convenience. Protein sources used in the formulation of the sample were different from chicken(C), salmon(S), poultry(P), fish(F), or turkey(T). Depending on the country of manufacture—Asian(A) or international(I), except for Asian countries and the source of protein—the samples were indicated as follows: ASC: Asian_salmon and chicken, AS: Asian_salmon, AP: Asian_poultry, AF: Asian_fish, AC: Asian_chicken, IS: International_salmon, ITS: International_turkey and salmon, IS2: Inter-national_salmon, IS3: International_salmon, and IS4: International_salmon. The biggest advantage of the E-tongue analysis system is that when analyzing numerous samples, data with objectivity and reproducibility can be obtained quickly [27]. The patterns of taste components and 10 types of dog food measured using an e-tongue are shown in Figure 1a,b. SRS (sourness) was the highest at 9.3 in ASC, followed by 8.8 and 7.4 in AF and ITS, respectively, and the lowest at 2.7 in AC. STS (saltiness) was the highest at 9.0 in AC, followed by 8.0 in IS3, and the lowest at 3.5 in AP. UMS (umami) is the highest at 8.6 in ASC and IS3 and the lowest at 2.0 in IS2. SWS (sweetness) is the highest at 7.6 in AC, followed by 7.5 in IS, respectively, and the lowest at 3.4 in IS4. BRS (bitterness) is the highest at 9.3 in AC, followed by 7.7 in IS and ITS, respectively, and the lowest at 2.7 in ASC.

Since dogs are unable to verbalize the palatability of pet food, it creates challenges for researchers to understand the acceptance and preference of dog foods from an animal’s point of view. In a way to overcome these difficulties, electronic tongue analysis with high objectivity and reproducibility is currently used in research on animal feed in addition to food consumed by humans [30]. ÉLES et al. used E-nose and E-tongue to classify commercial canned dog and cat foods, and it was found that joint application of E-nose and E-tongue analysis achieved the higher accuracy in discriminating canned pet foods containing different composition [30]. In addition, Cheli et al. suggested the use of electronic nose and tongue as rapid tools for evaluating and developing high quality pet foods [35]. In this present study, each of the 10 dry dog foods showed a unique taste profile. While international samples showed less variability in the pattern of taste attributes compared to domestic samples, any explainable taste pattern according to main protein sources and/or manufactures was not found. The results from the electronic tongue analysis alone may be limited to explain the flavor characteristics of samples. Thus, the integration of the results with other electronic sensors may provide useful information to understand the sensory properties of dog foods used in this study.

### 2.2. E-Nose Analysis

E-nose analysis is known to have the advantage of being able to identify volatile compounds with more objectivity than analysis by human smell, such as the aforementioned E-tongue analysis. E-nose analysis analyzes volatile compounds in many foods in a short period of time, makes it easy to derive results, and simultaneously analyzes individual volatile compounds and detects the overall pattern of volatile compound to provide results. Due to this convenience, the E-nose system is widely used to analyze the volatile compounds of food [27]. The volatile active compounds (VACs) in 10 types of dog food measured using an E-nose are shown in Supplementary Table S1. The compounds detected in the dog foods were 21 acids and esters, 24 alcohols, 16 aldehydes, 28 hydrocarbons, 18 heterocyclic compounds, 11 ketones, 5 furans, and 5 sulfur-containing compounds. A total of 34 VACs were detected in ASC, with higher contents of heterocyclic compounds than those of other compounds. A total of 31 VACs were detected in AS, with higher contents of furans than those of other samples. A total of 31 VACs were detected in AP, with higher contents of hydrocarbons than those of other compounds. A total of 28 VACs were detected in AF, with higher contents of aldehydes than those of other compounds. In AC, 26 VACs were detected, and the hydrocarbon content was higher than that of other compounds. In total, 29 VACs were detected in IS, with higher contents of heterocyclic compounds than those of other compounds. In total, 33 VACs with relatively high contents of alcohols were detected in the ITS. In total, 31 VACs were detected in IS2, with higher contents of hydrocarbons than those of other compounds. In total, 33 VACs were detected in IS3 and 24 VACs were detected in IS4, and a lot of heterocyclic compounds were detected in both samples.

Previous studies have suggested that odor has a great influence on the choice of dog food [36]. In particular, dog food is an interesting topic for studying volatile aroma compounds as its formulation is a complex mixture of grains, meat sources, and additives [32]. The volatile alcohols, which showed relatively high detection, were mainly formed from the oxidative decomposition of fat. The volatile compound of alcohols has been described as green, fruit, and flower scent [37]. Aldehyde is produced by lipid oxidation reactions and is used as an indicator of oxidation taste, and these aldehydes are detected with the volatile compound of various meat [38]. 2-Decenal detected only in IS4 was found to have a positive effect on dog food intake rates, while methional detected in ITS and IS3 were considered volatile compounds that negatively affect dog food intake rates [33]. In addition, pentanal detected in DP with poultry by-product dry powder and chicken oil was considered to be a volatile compound of chicken [38].

There is an animal panel for the development of pet foods, but various descriptions cannot be analyzed except for the degree of liking. Analysis of pet foods through the E-nose may be suitable for overcoming these limitations by being fast, reliable, and consistent. In this study, volatile compounds that may negatively affect food intake as well as components that may positively affect food intake were identified through the E-nose analysis of 10 dog foods. In a study by Oladipupo et al., the combination of an E-nose and an E-tongue were used to track animal acceptance and preference responses and were discovered as a way to develop new products with appropriate mixing ratios among different flavor formulations [31]. Furthermore, E-nose method classified commercial canned dog and cat foods by 95% and the highest discriminating results were achieved by the joint application of E-nose and E-tongue technology [30]. As studies have shown, the use of E-nose and E-tongue is suggested as a rapid screening tool for discriminating different aromas and tastes prior to traditional animal preference test in the development of new dog foods [39].

### 2.3. Analyses of Taste Components and Volatile Compounds Patterns via Multivariate Analyses

**Figure 2:**
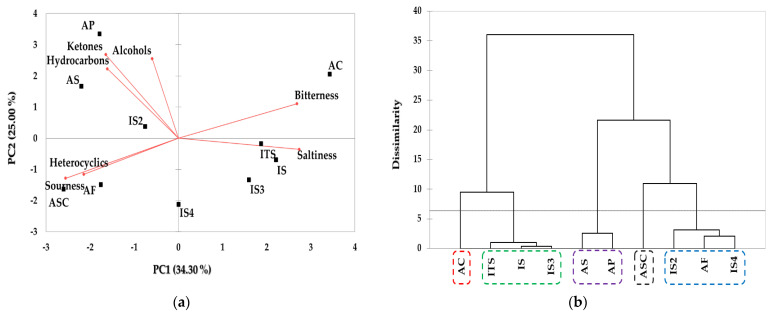
Taste components and volatile compounds by E−tongue and E−nose. (**a**) PCA (total variance 59.3%). (**b**) HCA. ASC: Asian_salmon and chicken, AS: Asian_salmon, AP: Asian_poultry, AF: Asian_fish, AC: Asian_chicken, IS: International_salmon, ITS: Internatonal_turkey and salmon, IS2: International_salmon, IS3: International_salmon, IS4: International_salmon.

The sensory characteristics of taste and volatile components in 10 dog foods analyzed by integrating the results of the E-tongue and E-nose analysis system were subjected to multivariate analysis using PCA and HCA. The results of PCA and HCA are shown in Figure 2a,b, respectively. Based on the sensory characteristics and patterns of the samples obtained via PCA, PC1 and PC2 contributed 34.30% and 25.00% of variance, respectively, accounting for 59.3% of the total variance. AC was influenced by bitterness and was located in the positive directions based on PC1 and PC2. Due to the influence of saltiness, ITS, IS, and IS3 were observed in the positive direction and negative directions based on PC1 and PC2, respectively. AP, AC, and IS2 were influenced by ketones, alcohols, and hydrocarbons and positioned in the second quadrant, while ASC and AF were affected by heterocyclics and sourness and located in the third quadrant. The results of HCA for the taste and volatiles compounds of dog foods are shown in Figure 2b, and five clusters representing the similarity between samples were identified. AC was identified as cluster 1 and cluster 2 included ITS, IS, and IS3. It was found that AS and AP were included in cluster 3 and ASC was identified as cluster 4. IS2, AF, and IS4 was classified into cluster 5. It was confirmed that cluster 3 showed the highest dissimilarity and cluster 2 showed a relatively low dissimilarity from the other groups.

Consistent with the individual results of the E-tongue and the E-nose, the multivariate analysis results did not show significant difference between domestic and international products or difference by protein source. Currently, multivariate analysis is used to evaluate dog foods in various research fields [9,40]. In a study by Alegría-Morán et al., the PCA of the 10-year food preference database was used to relate dogs’ food choices with nutritional composition of the diets, and it was found that the content of crude fiber and dry matter negatively associated with dogs’ food preferences [40]. The PCA analysis was also used to distinguish dog foods according to the similarity of the nutritional profile of the group [9]. Recent studies have shown that multivariate analysis of E-nose data can distinguish the aroma of beef and pork [41] and classify the aroma profile of beef according to quality grade [42]. In this study, dog foods were separated according to the sensory characteristics of taste (bitterness, saltiness, and sourness) and VACs through the multivariate analysis of chemical sensory analysis data. These results will provide fundamental information on developing new dog foods with improved palatability and on understanding the sensory characteristics of different varieties of dog food.

### 2.4. GC-MS Analysis

The VACs in 10 types of dog food measured using an GC-MS are shown in Supplementary Table S2. A total of 180 volatile compounds were detected in samples and were classified into 7 types, including 29 acids and ester, 19 alcohols, 17 aldehydes, 67 hydrocarbons, 21 heterocyclic compounds, 18 ketones, 7 furans, and 2 sulfur-containing compounds, respectively. In ASC sample, furans were detected the most and aldehydes were detected the most in AS and AP samples, among which hexanal content levels were found to be the highest. A relatively high content of hydrocarbon was detected in AF, while a relatively high content of aldehyde was detected in AC. The high contents of heterocyclics compounds were detected in IS, and ITS contained the high contents of hydrocarbons. It was also found that IS2, IS3, and IS4 contained hydrocarbons the most.

According to Koppel et al., aldehyde was identified as the most abundant volatile compounds in dry dog food, but this study found that the most abundant volatile compound was hydrocarbon in samples [32]. In this study, heptanal, benzaldehyde, octanal, nonanal, hexanal, pentanal, and decanal were detected as aldehydes, and in particular, nonanal was found in all samples and benzaldehyde detected in all samples except IS and ITS. Heptanal, benzaldehyde, octanal, and nonanal detected have been recognized as the key volatile compounds of dog food and were reported to be positively related to dog food intake [33,37]. Hexanal detected in ITS, AP, AF, AC, and IS4 has been reported as an aroma component of dry dog food in a recent study [37]. Pentanal detected in AS and decanal detected in ASC and AP were identified as volatile compound of chicken in cooked chicken, beef, and pork by-product blends [38]. Acids and ester, the second most abundant volatile compounds, were considered to exhibit some meat flavor in addition to the fruit flavor [38], and the hexanoic acid detected in ASC, ITS, and IS2 was considered as a positive flavoring compound in dog food [37].

### 2.5. GC-O Analysis

**Figure 3:**
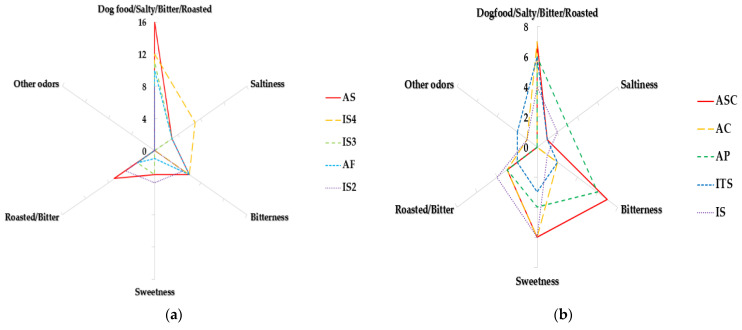
Aroma profiles in 10 dog foods using GC-O. (**a**) Strong dog food odor group. (**b**) Weak dog food odor group. ASC: Asian_salmon and chicken, AS: Asian_salmon, AP: Asian_poultry, AF: Asian_fish, AC: Asian_chicken, IS: International_salmon, ITS: International_turkey and salmon, IS2: International_salmon, IS3: International_salmon, IS4: International_salmon.

Analysis results of OACs and the aroma profiles in 10 dog foods using GC-O are shown in Table 1 and Figure 3a,b, respectively. The odor descriptor groups of the recognized OACs were divided into dog food, salty, bitter, roasted, sweet, and other odors. The group of dog food included aroma profiles with dog food, dog food/bitter, dog food/salty, and dog food/roasted. The other odor group was composed of alcohols, spicy, and sour descriptors. A total of 67 odor active compounds were detected in 10 dog foods. Among them, 23 compounds recognized as dog food odors are as follows: hexanoic acid, butanoic acid, 2-methyl-octanoic acid, pentanoic acid, propyl-propanedioic acid, 2-amino-4-methylbenzoic acid, 3-methoxy-propanal, heptanal, benzaldehyde, methylbenzene, 1,3-dimethyl-benzene, pulegone, sulfonyl-bis-methane, 2,2,4,6,6-pentamethylheptan, 3,3-dimethylhexane, dimethyl-4-aniline, dodecane, methoxy-phenyl-oxime, 2,6-dimethylpyrazine, 2-heptanone, dimethyl sulfone, 2-piperidinone, and furfural. Butanoic acid was detected in all except AC, and heptanal detected in AS, IS, IS3, and IS4 were recognized as a strong dog food scent.

Benzaldehyde recognized as dog food odor were detected in all samples except IS and ITS. Dodecan identified as a dog food odor was detected in AS, AP, AF, AC, IS3, and IS4, and it was recognized as a strong dog food odor in AS and AF. Nonanal detected in all samples was recognized as roasted odor, and 2-pentylfuran detected in all samples except IS3 and IS4 was identified as a sweet odor. The aroma profile was analyzed by dividing the 10 dog foods into 2 groups according to the intensity of the dog food odor. In five samples with strong dog food scent (AS, IS4, IS3, AF, and IS2), other scents were recognized as relatively low intensity, and the plot clearly showed that dog food odor is dominant over other aroma profiles (Figure 3a). ASC, AC, AP, ITS, and IS, which were recognized as relatively weak dog food scent, showed less variable in the strength of aroma notes and the plot of these five samples demonstrated that the intensity of bitter and sweet odors was increased compared to those with strong dog food odor groups (Figure 3b).

It was reported that hexanoic acid, heptanol, benzaldehyde, and 2-heptanone, which were described as dog food odor in this study, were correlated with the palatability of dog foods. In particular, the addition of benzaldehyde to the basal dog food significantly increased the consumption ratio, indicating an improvement in palatability of dog food [37]. As in this study, nonanal representing roasted and bitter have been found in all samples in the other literature [32], suggesting that nonanal may be a common volatile compound contributing to the odor of dog food. Benzeneacetaldehyde, detected in AS, IS, and IS2, was recognized as a sweet odor. 2-Pentylfuran was detected in all samples except IS3 and IS4 and was also characterized by sweet odor [37]. Recent studies have shown that octanal, nonanal, 2-pentylfuran, and heptanal were identified as volatile compounds associated with intake ratio of dog food and the addition of these compounds to odorless basal dog food significantly increased consumption ratio [33]. Therefore, octanal, nonanal, 2-pentylfuran, heptanal, and benzaldehyde may have a positive effect on food intake as OACs in dog food used in this experiment.

### 2.6. Analyses of Odor Active Compound Patterns via Multivariate Analyses

**Figure 4:**
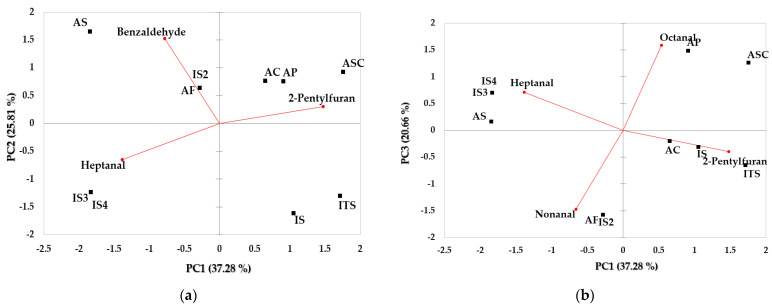
PCA plot for key odor compounds by GC-O. (**a**) PC1−2 (total variance 63.09%) and (**b**) PC1−3 (total variance 57.94%). ASC: Asian_salmon and chicken, AS: Asian_salmon, AP: Asian_poultry, AF: Asian_fish, AC: Asian_chicken, IS: International_salmon, ITS: International_turkey and salmon, IS2: International_salmon, IS3: International_salmon, IS4: International_salmon.

**Figure 5:**
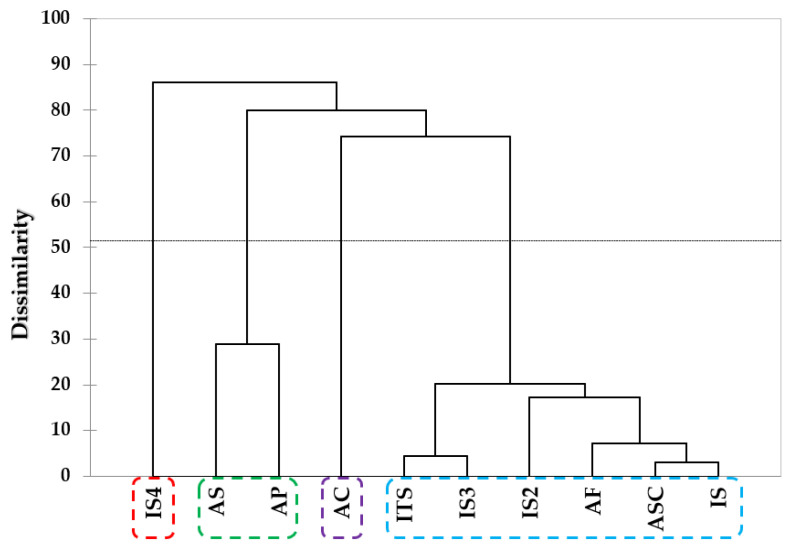
Cluster for key odor compounds of 10 dog foods by GC-O. ASC: Asian_salmon and chicken, AS: Asian_salmon, AP: Asian_poultry, AF: Asian_fish, AC: Asian_chicken, IS: International_salmon, ITS: International_turkey and salmon, IS2: International_salmon, IS3: International_salmon, IS4: International_salmon.

Each sample was separated through principal component analysis and cluster analysis using the results of the odor active compounds in 10 dog foods identified via GC-O, and the results of PCA and HCA are shown in Figure 4a,b and Figure 5, respectively. The PCA plot revealed that the samples were distinguished according to key odor active compounds which may be related to the increase in palatability of dog foods. Key odor active compounds used for PCA analysis included octanal, nonanal, 2-pentyl furan, heptanal, and benzaldehyde.

The three components PC 1, PC 2, and PC 3 explained 37.28%, 25.81%, and 20.66% of the total data variance, respectively, amounting for 83.75% of cumulative variance (Figure 4). Figure 4 shows that AC, AP, ASC, IS, and ITS were positioned in the positive (+) direction based on PC1 which was positively associated with 2-pentylfuran and octanal. It is observed that AS, AF, IS2, IS3, and IS4 were located in the negative (−) direction based on PC1 which was negatively related with benzaldehyde, heptanal, and nonanal. In PC1–2, AS, IS2, and AF were affected by benzaldehyde and positioned in the second quadrant representing negative PC1 and positive PC2. In PC1–3, AP and ASC affected by octanal were identified in the first quadrant, and AC, IS, ITS were identified in the fourth quadrant. AF and IS2 influenced by nonanal were located in the third quadrant, corresponding to negative values of PC1 and PC3.

The results of cluster analysis using the OACs dataset for 10 dog foods classified four clusters representing dissimilarity between samples. IS4 was identified as cluster 1, and cluster 2 included AS and AP. It was found that AC was identified as cluster 3, and cluster 4 included ITS, IS3, IS2, AF, ASC, and IS. It was confirmed that cluster 1 showed the highest dissimilarity and cluster 4 showed a relatively low dissimilarity from the other groups. Similar to the results of the electronic sensors, the multivariate analysis of OACs did not demonstrate any significant differences between manufacturers (domestic or international) or protein sources.

GC-O is an analytical method for detecting the active compounds of food odor using human nose via an olfactory port and has been widely used in various fields of food analysis research [43]. Aceña et al. used head space solid-phase microextraction and GC-O techniques to identify the key odorants in commercial sherry vinegar and calculated odor activity value (OAV) to assess the contribution of the most important aromatic compounds found to overall sherry vinegar aroma [44]. In a study by Yu et al., a total of 27 odor active compounds were detected in 4 commercially available oyster sauces using GC-O and the samples were classified according to different aroma profiles through the PCA analysis [45]. Ten commercially available dog foods used in this study were separated according to key OACs (octanal, nonanal, 2-pentyl furan, heptanal, benzaldehyde) through the multivariate analysis. Since the samples used in this experiment contained various ingredients including different protein sources and produced in different manufacturers, dog foods may not have been clearly separated by PCA and HCA using the dataset of the OACs identified via GC-O. In addition, further study is needed to investigate whether the major aromatic compounds identified in this study affect the increase in the palatability of dry dog food. However, volatile compounds responsible for the characteristic aroma of commercial dog foods were assessed using GC-O and multivariate analysis in the present study for the first time. These results are expected to be used as primary data for aroma evaluation in producing new dog foods with improved palatability.

## 3. Materials and Methods

### 3.1. Materials

Overall, 10 commercially available dry dog foods from different manufacturers were purchased from online stores. The selection criteria for the dog foods were that they had to be popular, highly rated (best-selling), and dry dog food. Protein sources used in the formulation of the sample were different from chicken(C), salmon(S), poultry(P), fish(F), or turkey(T). Depending on the country of manufacture—Asian(A) or international(I), except for Asian countries and the source of protein—the samples were indicated as follows: ASC, AS, AP, AF, AC, IS, ITS, IS2, IS3, IS4. The samples were ground to the same size to minimize the error due to size difference and stored at 4 °C before use.

### 3.2. Electronic Tongue (E-Tongue) Analysis

Taste properties in dog foods were measured using E-tongue (ASTREE, Alpha MOS, Toulouse, France). The E-tongue consists of a multi-channel electrode with a converter composed of a lipid membrane fixed with a polymer for the purpose of mimicking the function of a human taste receptor and is used to measure five taste properties. The measured potential in the E-tongue is used to determine the concentration of some components of the solution. These E-tongues are easy to apply to liquid foods, and the preparation is limited to dilution or filtration [26]. The sample for E-tongue analysis was made by mixing between dog food 5 g and purified water 100 mL for 20 min at 300 rpm. In total, 10 mL of filtered solution was mixed with 90 mL of purified water and was used for E-tongue analysis. The analysis of taste component was performed after contacting the electronic tongue sensor for 120 s. The module in E-tongue includes five basic taste sensors, sourness (SRS), saltiness (STS), umami (UMS), sweetness (SWS), and bitterness (BRS), and two reference sensors with an attached reference electrode (Ag/AgCl) which includes the calibration components of metallic taste (GPS) and spiciness (SPS). The taste sensor responses in E-tongue were converted to taste scores ranged from 1 to 12. The sensors were cleaned after each measurement using purified water in order to prevent errors in the analysis results due to cross-contamination between samples. Taste component patterns between sample groups were confirmed through principal component analysis (PCA) and hierarchical cluster analysis (HCA) [46].

### 3.3. Electronic Nose (E-Nose) Analysis

Volatile compounds in dog foods were measured using E-nose (HERACLES Neo, Alpha MOS, Toulouse, France). The E-nose is a technology designed to imitate the olfactory system of human beings. In addition, the E-nose is a device consisting of an electrochemical sensor array with partial specificity and an appropriate pattern recognition system that can recognize simple or complex smells [26]. The dog food 2 g was placed in the headspace vial (22.5 × 75 mm, PTFE/silicone septum, aluminum cap), and the volatile compounds were collected in the headspace during the stirring process at 500 rpm for 20 min at 50 °C in E-nose system incubator. After the collection process, volatile compounds (2000 μL) were collected by an automatic sampler which was attached to E-nose system and into the injector. The flow rate of hydrogen gas was set to 1 mL/min. For the acquisition of volatile compounds, the time was 277 s for each sample, and trap absorption and trap desorption temperature for volatiles was 40 °C and 250 °C, respectively. Oven temperature was set to 40 °C for 5 s, elevated to 270 °C at a rate of 4 °C/s, and kept for 30 s. To identify each volatile, Kovat’s index library-based AroChembase (Alpha MOS) was used. Volatile compounds patterns between sample groups were confirmed through PCA and HCA [46].

### 3.4. GC-MS Coupled with GC-Olfactometry (GC-O)

In order to collect volatile compounds contained in dog food, a headspace analysis method using SPME (Supelco Inc., Bellefonte, PA, USA) coated with 50/30 m, DVB/CAR/PDMS was used. Two grams of sample were placed in a headspace vial and sealed with an aluminum cap, and then the vial was heated for 10 min at 60 °C to reach its equilibrium. After the equilibration, the SPME (solid-phase microextraction) fiber was injected into the vial and the volatile compounds were absorbed by the fiber for 30 min at 60 °C. Volatile compounds in dog foods were analyzed using gas chromatography–mass-spectrometry (GC-MS: Agilent 7890A & 5975C, Agilent Technologies, Santa Clara, CA, USA) and HP-5MS column (30 m × 0.25 mm i.d. × 0.25 um film thickness). For the GC-MS analysis conditions, the oven temperature was at 40 °C for 5 min and then accelerated to 200 °C at a rate of 5 °C/min. The injector temperature was 220 °C, the flow rate of helium was 1.0 mL/min, and the split ratio was 1:10. Each compound separated from the total chromatogram (TIC) was sympathetic to the mass spectrum library (NIST 12) and the literature, and each concentration of volatile compound was calculated by converting the peak area of the internal standard material (pentadecane) into a peak area of μg/g. Odor active compounds (OACs) were measured using GC-MS coupled with GC-O (olfactometry detection port III (ODP-III), Gerstel Co., Linthicum, MD, USA). Identification of odor active compounds in dog foods were performed 20 min (5–25 min) in odor to solvent elution time (5 min) and general detected time of OACs. The intensity of OACs was expressed as 4 levels ranged from 1 to 4, and a higher number indicates higher odor active level [27].

### 3.5. Statistical Analysis

PCA and HCA applied for multivariate analysis used XLSTAT software ver. 9.2 (Addinsoft, New York, NY, USA) to identify how dog food was located in the pattern of chemical sensory properties. PCA creates a new orthogonal axis or set of variables from the original variable, known as the principal component (PC). Each PC is defined as a vector known as an eigenvector of the variance-covariance matrix, and the variance that follows the vector is called an eigenvalue. Based on Kaiser rules, the PCA displays the variables and samples mapped through the loading and scoring of dimensional spaces determined by the PC with eigenvalues greater than 1.0 based on a Kaiser’s rule [47]. Hierarchical cluster analysis (HCA) was performed to identify relative dissimilarity among samples and reported as a dendrogram. Each dissimilarity in the dendrogram was achieved on the basis of the Euclidean distance between samples using Ward’s algorithm as the agglomerative method. The hierarchical algorithm constructs the nested grouping of patterns and similarity levels at which groupings change [48].

## 4. Conclusions

In this study, the sensory characteristics of taste and volatile compounds in 10 commercially available dry dog foods were evaluated using electronic sensors. A total of 10 dog foods were separated according to the sensory properties of taste (bitterness, saltiness, and sourness) and VACs through the multivariate analysis of integrated results of the E-tongue and E-nose analyses. In this study, E-nose provides the relative similarity of the sample group through the overall pattern of total compounds rather than individual compounds. A total of 67 odor active compounds were detected in 10 dog foods through GC-MS/O, of which 23 compounds were recognized as dog food odors. GC-MS identifies substances by fragmentation patterns and provides more objective information about individual compounds. The aroma profile was analyzed by dividing the samples into two groups depending on the intensity of the dog food odor, and it was demonstrated that the dog food odors were dominant over other odors in the sample groups with relatively strong intensity of dog food scent. Among odor active compounds detected via GC-O analysis in 10 dog foods, octanal, nonanal, 2-pentyl furan, heptanal, and benzaldehyde were identified as key odor compounds. Dog food samples were classified according to key odor active compounds through the multivariate analysis.

The identification of key odor active compounds in commercially available dry dog foods presented in this study is required for understanding the effect of flavor attributes of dog foods on overall palatability. However, further study is needed to validate whether these key OACs affect the increase in the palatability of dry dog food through preference test and/or acceptance tests. Despite these research limitations, to the best of our knowledge this is the first study to assess the volatile active compounds properties of dry dog foods using GC-MS/O. The results of this present study will provide fundamental information on producing a new dog food with improved palatability. These results are also expected to be used as basic data for establishing a standardized analysis system needed for new product development and quality management in a wide variety of pet food industries.

## Figures and Tables

**Table 1:** Odor description and intensity in dog food using GC-Olfactometry.

Volatile Compounds	RT ^(1)^	Relative Intensity										Odor Description
	(min)	ASC	DS	DP	DF	DC	IS	ITS	IS2	IS3	IS4	
**Acids and esters (14)**												
Hexanoic acid	7.65	1	0 ^(2)^	0	0	0	0	1	2	0	0	Dogfood
Butanoic acid	8.143	2	1	0	2	0	1	1	2	1	1	Dogfood, Bitter
3-Methyl-butanoic acid	9.594	0	2	0	1	0	1	1	0	1	1	Salty
Isovaleric acid	9.765	0	0	0	0	0	0	0	0	0	1	Salty
2-Methyl-octanoic acid	10.07	0	1	0	0	0	0	1	0	0	1	Dogfood
2-Methyl-butanoic acid	10.086	0	1	2	0	0	1	0	0	2	1	Salty
Pentanoic acid	11.005	0	1	0	1	0	0	2	1	0	0	Salty, Dogfood
Propyl-propanedioic acid	11.112	0	0	0	0	0	1	0	0	0	0	Dogfood
2-Amino-4-methylbenzoic acid	11.409	0	0	0	0	0	0	0	0	1	1	Dogfood
4-Methyl-pentanoic acid	12.873	1	0	1	0	0	0	0	0	0	1	Salty
Heptanoic acid	14.084	1	1	1	2	0	1	0	1	0	1	Bitter
Sobutyl octyl carbonate	15.261	0	0	0	0	0	0	0	1	0	0	Roasted
Sorbic Acid	16.864	0	0	1	0	0	0	0	0	0	0	Bitter
Heptanoic acid, ethyl ester	17.545	2	0	0	0	0	0	0	0	0	0	Bitter
**Alcohols (5)**												
Heptanol	13.52	0	0	1	0	0	0	0	0	0	0	Bitter
1-Octen-3-ol	13.839	0	2	1	0	0	0	0	0	0	0	Bitter
4-Methyl-phenol	16.815	0	0	0	0	0	1	0	0	0	0	Alcohols
9-Octadecen-1-ol	18.722	0	0	0	0	1	0	0	0	0	0	Sweet
4-Terpineol	19.938	0	0	0	0	0	0	1	0	0	0	Spicy
**Aldehydes (8)**												
3-Methoxy-propanal	7.717	0	0	0	0	0	0	0	1	0	0	Dogfood
Hexanal	7.951	0	1	2	1	1	0	0	0	0	1	Bitter
Heptanal	11.27	0	2	0	0	0	1	0	0	2	2	Dogfood
Benzaldehyde	13.217	1	2	1	1	2	0	0	1	1	1	Dogfood, Bitter
Octanal	14.587	2	1	2	0	0	0	0	0	0	0	Sweet
2,4-Heptadienal	14.834	0	0	0	0	1	0	0	0	0	0	Sweet
Benzeneacetaldehyde	15.9	0	1	0	0	0	2	0	1	0	0	Sweet
Nonanal	17.72	1	2	1	2	1	1	1	2	1	1	Roasted, Bitter
**Hydrocabons (21)**												
Methylbenzene	6.991	0	1	0	1	0	0	1	0	0	1	Bitter, Dogfood
Ethylbenzene	9.932	0	0	0	0	1	0	0	0	0	0	Sour
4-Methyl-octane	9.992	2	0	0	0	0	0	0	2	0	0	Bitter
1,4-Dimethyl-benzene	10.177	0	0	0	2	0	0	0	0	0	0	Salty
1,3-Dimethyl-benzene	10.262	0	2	0	2	0	0	0	0	0	0	Dogfood, Roasted
Pulegone	11.637	0	0	1	0	0	0	0	0	0	0	Dogfood
Sulfonylbis-methane	11.808	0	0	0	0	1	0	0	0	0	0	Dogfood
2-Decene	13.648	0	0	0	1	0	0	0	0	0	0	Bitter
2,2,4,6,6-Pentamethylheptan	14.176	0	0	0	0	0	0	0	0	2	2	Dogfood, Bitter
3,3-Dimethylhexane	15.563	0	0	0	0	0	0	0	0	0	1	Dogfood
2-Methyldecane	16.19	0	0	0	2	0	0	2	1	0	2	Bitter
2,2,3,4-Tetramethylpentane	16.269	0	0	1	0	0	0	0	0	0	0	Roasted
3-Methylhexadecane	16.476	0	0	1	0	0	0	0	0	0	0	Sweet
3,8-Dimethyldecane	16.493	0	0	0	0	0	0	0	1	0	0	Sweet
Undecane, 2,8-dimethyl-	16.867	0	0	0	0	0	0	1	0	0	1	Roasted
2,3,6,7-Tetramethyloctane	17.107	0	0	0	0	1	0	0	1	1	0	Sweet
3,3-Dimethylhexane	17.266	0	0	0	0	1	0	0	1	0	0	Bitter
Dimethyl-4-aniline	18.001	0	0	0	0	0	0	0	0	2	0	Dogfood
2,3,5-Trimethyldecane	18.877	0	0	0	0	0	0	0	0	0	1	Salty
2-Butyl-1-decene	19.547	0	0	0	0	0	0	0	0	0	1	Roasted
Dodecane	20.472	0	2	1	2	1	0	0	0	1	1	Dogfood, Salty
**Heterocyclic compounds (8)**												
Methoxy-phenyl-oxime	11.343	0	0	0	0	1	0	0	0	0	0	Dogfood
2,6-Dimethylpyrazine	11.603	1	2	2	0	0	1	0	0	0	0	Dogfood
2-Acetylpyrrole	16.397	0	0	0	0	0	1	0	0	1	0	Sweet
2,6-Diethylpyrazine	16.95	0	0	0	0	0	0	0	0	1	0	Sweet
2-Ethyl-3,5-dimethyl-pyrazine	16.971	1	2	1	1	1	2	0	0	0	0	Roasted, Bitter
Tetramethyl-pyrazine	17.213	0	1	0	0	0	0	0	0	0	0	Bitter
2-3-Indole	18.995	1	2	0	0	0	1	0	2	2	0	Roasted, Bitter
1-2-Pyrrolidon	20.862	0	0	0	0	0	0	0	0	0	2	Bitter
**Ketones (8)**												
2-Heptanone	10.952	0	0	1	0	1	0	0	0	0	0	Dogfood
Dimethyl sulfone	11.827	0	0	0	0	1	0	0	0	0	0	Dogfood
6-Methyl-5-hepten-2-one	14.055	0	0	0	0	0	0	0	0	0	2	Salty
3,5-Octadiene-2-one	16.71	0	0	0	0	1	1	1	0	0	0	Sweet
2-Nonanone	17.362	2	1	0	0	0	0	0	0	0	0	Bitter
5-Methyl-2-cyclohexanone	19.568	0	0	0	0	0	0	1	0	0	0	Spicy
2-Piperidinone	19.813	0	2	0	1	0	0	0	0	1	1	Dogfood
Chrysanthenone	20.88	0	1	0	0	0	0	0	0	0	0	Roasted
**Furans (3)**												
Furfural	9.153	2	0	0	0	0	0	0	1	0	0	Dogfood
2-Furanmethanol	9.758	2	0	0	0	0	0	0	0	0	0	Sweet
2-Pentylfuran	14.219	2	1	1	1	2	2	2	1	0	0	Sweet

ASC: Asian_salmon and chicken, AS: Asian_salmon, AP: Asian_poultry, AF: Asian_fish, AC: Asian_chicken, IS: International_salmon, ITS: International_turkey and salmon, IS2: International_salmon, IS3: International_salmon, IS4: International_salmon. ^(1)^ RT: retention time ^(2)^ 0 corresponds ‘not recognized’.

## Data Availability

All data are provided in the article.

## Supplementary Materials

The following supporting information can be downloaded at: https://www.mdpi.com/article/10.3390/molecules28145509/s1. Table S1: Volatile compounds of 10 dog foods using E-nose; Table S2: Volatile compounds of 10 dog foods using GC/M.

## Sample Availability

Samples of the compounds are not available form the authors.
