# Supplementary material for: Evaluation of the Chemosensoric Properties of Commercially Available Dog Foods Using Electronic Sensors and GC-MS/O Analysis
Source: Molecules. 2023 Jul 19;28(14):5509. doi: 10.3390/molecules28145509 (PMC10384845; doi:10.3390/molecules28145509)
Supplement: Supplementary file 1 [file molecules-28-05509-s001.zip › molecules-2473744-SI.pdf]

Supplementary Material

Evaluation of the Chemosensoric Properties of Commercially Available Dog Foods using Electronic Sensors and GC-MS/O Analysis

Hyangyeon Jeong[1†], Moon Yeon Youn[2†], Sojeong Yoon[1], Seong Jun Hong[1], Seong Min Jo[1], Kyeong Soo Kim[3], Eun Ju Jeong[4], Hyun-Wook Kim[5], Eui-Cheol Shin[1,2,6*]

1. Supplementary Tables

Supplementary Table S1 Volatile compounds of 10 dog foods using E-nose.

(Peak area x 10^3)

Compounds	RT[1)](RI[2)])	Sensory description	ASC	AS	AP	AF	AC	IS	ITS	IS2	IS3	IS4
**Acids and esters(21)**												
Methyl formate	17.58(380)	Fruity	10.16±0.82	12.98±0.60	0.28±0.03	8.15±1.12	ND[3)]	ND	ND	ND	ND	ND
Methyl acetate	27.21(533)	Fruity	ND[3)]	ND	2.78±0.34	ND	ND	ND	ND	ND	ND	ND
Formic acid	29.78(574)	-	ND	0.53±0.04	ND	ND	ND	ND	ND	ND	ND	ND
Acetic acid	33.29(616)	Sour	ND	ND	0.13±0.03	ND	ND	ND	ND	ND	ND	ND
Isopropyl acetate	36.31(641)	Sweet	ND	ND	0.05±0.09	ND	ND	ND	ND	ND	ND	ND
Propyl acetate	45.21(718)	Sweet	ND	ND	ND	ND	ND	ND	ND	ND	0.25±0.06	ND
Methyl butanoate	45.24(719)	Sweet	0.14±0.03	ND	ND	ND	0.06±0.01	ND	ND	ND	ND	ND
Butyl acetate	54.13(804)	-	ND	ND	ND	ND	ND	ND	ND	ND	ND	0.21±0.02
Propyl propanoate	54.25(806)	Fruity, Sweet	ND	ND	ND	ND	ND	ND	0.18±0.05	ND	ND	ND
Propylacrylate	55.73(823)	-	ND	ND	ND	0.10±0.01	ND	ND	ND	ND	ND	ND

Methyl pentanoate	55.81(824)	Fruity, Nutty	ND	ND	ND	ND	ND	ND	0.08±0.01	ND	ND	ND
Ethyl 2-methylbutyrate	57.87(848)	Sweet	ND	ND	ND	ND	ND	ND	ND	ND	ND	0.16±0.01
Isoamyl acetate	59.29(865)	Fruity, Fresh	ND	ND	ND	ND	ND	ND	ND	0.02±0.03	ND	0.09±0.03
Pentanoic acid	61.95(897)	Beefy. Sweet	ND	ND	ND	1.05±0.41	1.32±0.06	ND	ND	ND	ND	ND
Heptanoic acid	71.69(1,142)	Cheese, Oily	ND	ND	ND	ND	ND	ND	ND	0.07±0.02	ND	ND
Benzoic acid	71.80(1,143)	-	ND	0.06±0.05	ND	ND	ND	ND	ND	ND	ND	ND
Benzyl acetate	73.13(1,166)	Burnt, Sweet	ND	0.11±0.10	ND	ND	ND	ND	0.10±0.06	ND	ND	ND
Methyl cinnamate	85.44(1,399)	Fruity, Sweet	0.19±0.04	0.21±0.01	0.17±0.02	ND	ND	ND	ND	ND	ND	ND
Methyl dodecanoate	91.40(1,531)	Coconut, Oily	0.64±0.04	ND	ND	ND	0.11±0.01	ND	ND	ND	ND	ND
Octyl caprylate	101.51(1,761)	Oily	ND	ND	ND	0.09±0.01	ND	0.09±0.02	0.09±0.01	ND	ND	ND
Dodecyl butyrate	101.66(1,765)	Oily, Fruity	0.10±0.03	ND	ND	ND	1.69±0.09	ND	ND	ND	ND	ND
**Alcohols(24)**												
Methanol	20.33(423)	-	ND	4.17±0.44	ND	3.20±0.18	2.15±0.14	3.33±0.41	4.89±0.19	2.00±0.18	ND	ND
Ethanol	21.21(437)	Ethanol, Sweet	ND	1.09±0.04	ND	ND	0.28±0.05	0.89±0.05	1.86±0.01	ND	ND	ND
2-Methyl-2-propanaol	23.67(476)	-	ND	ND	ND	ND	ND	0.14±0.09	0.56±0.04	0.29±0.06	0.06±0.10	ND
2-Propanol	25.17(500)	Etheral, Acetone	ND	ND	2.74±0.74	ND	ND	ND	ND	ND	ND	ND
1-Propanol	27.27(534)	Ethanol, Fruity	1.74±0.24	ND	ND	ND	ND	ND	2.16±0.21	1.60±0.18	6.30±0.68	1.92±0.29
2-Mercaptoethanol	29.63(571)	Sulfurous	ND	ND	ND	ND	ND	ND	ND	0.64±0.21	ND	ND
2-Butanol	29.87(575)	Oily	0.42±0.17	ND	ND	ND	2.66±0.07	ND	ND	ND	ND	ND
2-Methyl-1-propanol	33.19(615)	Bitter, Oily	ND	ND	ND	ND	ND	0.14±0.02	ND	ND	ND	ND

Butanol	39.81(671)	Oily, Sweet	0.12±0.11	ND	0.76±0.14	ND	ND	ND	ND	ND	ND	ND
1-Methoxy-2-propanol	40.39(675)	-	ND	ND	ND	ND	ND	ND	1.75±0.21	ND	1.39±0.24	ND
1-Penten-3-ol	40.42(676)	Burnt, Butter	ND	7.26±0.35	ND	ND	ND	ND	ND	ND	0.10±0.17	ND
Trometamol	41.43(684)	Burnt, Butter	ND	ND	ND	ND	ND	ND	ND	0.07±0.06	ND	ND
3-Pentanol	43.56(702)	Nutty, Oily	3.83±0.27	ND	12.18±1.46	1.35±0.07	2.01±0.13	0.07±0.01	ND	ND	ND	ND
3-Methyl-1-butanol	47.63(741)	Bitter, Butter	ND	0.16±0.01	ND	0.29±0.08	ND	ND	ND	ND	ND	ND
1,2-Butanediol	49.66(761)	Butter	0.57±0.03	ND	0.10±0.01	ND	0.09±0.01	ND	ND	ND	0.03±0.04	ND
Pentanol	49.70(761)	Fruity, Oily	ND	0.09±0.05	ND	ND	ND	ND	ND	ND	ND	ND
1-Hexen-3-ol	50.94(773)	Green	0.47±0.09	ND	ND	ND	0.24±0.02	ND	ND	ND	ND	ND
2,3-Butanediol	53.12(794)	Fruity	0.14±0.03	0.13±0.00	ND	0.28±0.03	ND	ND	ND	ND	ND	ND
2-Hexanol	54.20(805)	Fruity	0.59±0.05	0.56±0.01	0.54±0.04	ND	0.20±0.02	0.24±0.06	ND	0.20±0.03	ND	ND
2,3-Dimethyl-1-pentanol	55.73(823)	-	ND	ND	ND	ND	ND	0.16±0.01	ND	0.14±0.01	0.25±0.01	ND
1-Hexanol	59.45(867)	Oily, Sweet	ND	ND	ND	ND	ND	ND	0.11±0.01	ND	ND	ND
2-Cresol	67.73(1,062)	Sweet	ND	ND	ND	ND	ND	ND	ND	ND	0.78±0.13	ND
α-Terpineol	74.85(1,194)	Citrus, Oily	ND	ND	ND	ND	ND	ND	0.09±0.01	ND	ND	ND
2-Butyloctanol	79.90(1,288)	-	ND	0.28±0.07	ND	ND	ND	ND	ND	ND	ND	ND
**Aldehydes(16)**												
Propenal	22.58(459)	Almond	ND	2.83±0.13	ND	ND	ND	ND	ND	ND	ND	ND
Propanal	24.81(495)	Cocoa, Nutty	ND	ND	ND	ND	ND	ND	0.23±0.05	ND	ND	ND
Butanal	30.15(579)	Cocoa, Musty	ND	ND	ND	5.45±0.23	ND	1.18±0.05	1.80±0.02	ND	3.29±0.19	2.15±0.09
2-Butenal	35.98(638)	Green	2.28±0.04	0.16±0.12	0.10±0.06	0.09±0.01	ND	0.15±0.02	ND	0.23±0.05	ND	ND
2-Methylbutanal	38.63(661)	Almond, Coffee	ND	ND	ND	ND	ND	ND	ND	ND	0.62±0.00	ND

Pentanal	42.43(693)	Almond, Green	ND	ND	0.51±0.16	ND	ND	ND	ND	ND	ND	ND
2-Pentenal	47.70(742)	Oily, Green	0.35±0.04	ND	ND	ND	ND	ND	ND	ND	ND	ND
2-Methylpentanal	49.53(760)	Cheese, Fruity	ND	ND	ND	ND	0.06±0.00	ND	ND	ND	ND	0.06±0.01
2-Hexenal	57.98(850)	Almond, Oily	ND	0.07±0.01	ND	ND	ND	ND	ND	ND	ND	ND
2-Nonenal	71.71(1,142)	Citrus, Oily	ND	ND	ND	ND	0.04±0.03	ND	ND	ND	ND	ND
6-Decenal	75.26(1,201)	Green	ND	0.09±0.04	ND	ND	ND	ND	ND	ND	ND	ND
2-Decenal	78.75(1,266)	Green	ND	ND	ND	ND	ND	ND	ND	ND	ND	0.03±0.03
α-Terpinen-7-al	78.79(1,267)	Oily	ND	ND	0.02±0.03	0.04±0.04	ND	0.02±0.03	ND	ND	0.02±0.03	ND
2,4-Decadienal	79.89(1,287)	Oily, Green	ND	ND	0.27±0.05	0.31±0.02	ND	0.28±0.05	ND	ND	0.29±0.07	ND
Undecanal	80.68(1,302)	Citrus, Oily	0.34±0.04	ND	ND	ND	ND	ND	ND	ND	ND	0.27±0.07
Dodecanal	85.81(1,407)	Citrus, Oily	ND	ND	ND	0.17±0.01	ND	ND	ND	ND	ND	ND
**Hydrocarbons(28)**												
Butane	17.61(380)	-	0.51±0.37	ND	29.92±3.81	ND	ND	ND	ND	ND	ND	ND
2-Methylbutane	22.55(459)	-	ND	ND	ND	3.45±0.48	ND	ND	ND	10.06±1.61	ND	ND
Dichloromethane	24.77(494)	Sweet	ND	0.26±0.06	ND	ND	5.16±0.46	ND	ND	ND	0.36±0.33	ND
Acetonitrile	27.27(534)	Sweet	4.05±0.38	6.80±0.13	ND	2.44±0.24	ND	ND	ND	ND	ND	ND
3-Methylpentane	29.82(574)	-	ND	ND	0.30±0.11	ND	1.55±0.02	ND	ND	ND	ND	ND
Hexane	31.49(600)	Alkane	1.59±0.24	ND	ND	ND	0.02±0.03	1.02±0.10	1.59±0.01	ND	1.65±0.09	2.21±0.15
1,2-Dichloroethene	32.91(612)	Sweet	ND	ND	ND	ND	ND	ND	ND	ND	ND	ND
Benzene	37.98(655)	-	0.43±0.03	ND	ND	ND	ND	ND	ND	ND	ND	ND
Trichloroethylene	42.36(692)	Sweet	ND	0.18±0.05	ND	ND	ND	ND	ND	ND	ND	ND
Heptane	42.41(692)	Alkane	ND	ND	ND	ND	ND	ND	0.37±0.13	ND	ND	ND

1,2-Dichloropropane	43.55(702)	Sweet	ND	ND	ND	ND	ND	ND	2.47±0.14	ND	1.09±0.04	ND
Dibromomethane	43.65(703)	-	ND	ND	ND	ND	ND	1.00±0.12	ND	ND	ND	ND
Ethylcyclopentane	46.81(734)	-	ND	ND	ND	ND	ND	ND	ND	ND	0.32±0.24	ND
Methylcyclohexane	47.35(739)	Sweet	ND	ND	ND	ND	ND	0.08±0.08	ND	ND	ND	ND
4-Octene	53.11(794)	-	ND	ND	0.09±0.00	ND	ND	0.06±0.01	0.06±0.05	ND	0.24±0.03	ND
2,4-Octadiene	55.83(824)	-	ND	ND	0.13±0.01	ND	0.06±0.01	ND	ND	ND	ND	ND
Chlorobenzene	57.95(849)	Almond	ND	ND	0.06±0.01	ND	ND	0.14±0.02	ND	ND	ND	ND
Nonane	61.95(897)	Alkane	ND	1.57±0.17	1.36±0.12	ND	2.64±1.20	0.52±0.10	ND	0.80±0.25	ND	1.35±0.42
Decane	65.25(998)	Sweet, Fruity	3.90±3.13	3.51±2.54	3.15±1.66	3.00±1.33	ND	2.34±1.16	2.52±0.94	2.06±0.82	2.26±0.73	2.17±0.72
Limonene	67.23(1,050)	Citrus	1.13±0.51	1.01±0.40	1.16±0.37	1.33±0.64	ND	ND	ND	ND	ND	ND
γ-Terpinene	67.33(1,052)	Citrus, Oily	ND	ND	ND	ND	ND	0.29±0.23	1.08±0.29	ND	ND	0.94±0.29
1,2-Dihydrobenzene	67.35(1.053)	-	ND	ND	ND	ND	ND	ND	ND	0.72±0.40	ND	ND
5-Ethyldecane	71.68(1,141)	-	0.04±0.03	ND	ND	0.08±0.03	0.06±0.06	ND	ND	ND	ND	ND
Diterpene	73.36(1,169)	-	0.10±0.02	ND	ND	ND	0.16±0.00	ND	ND	ND	ND	ND
Tetradecane	85.71(1,405)	Alkane	ND	ND	ND	ND	0.60±0.03	0.16±0.01	ND	0.16±0.03	0.18±0.01	ND
2,4-Dinitrotoluene	91.28(1,529)	-	ND	0.68±0.03	0.66±0.04	ND	ND	ND	ND	ND	ND	ND
2-Methylheptadecane	101.65(1,764)	-	ND	ND	ND	ND	ND	ND	ND	0.10±0.01	0.11±0.01	ND
**Heterocyclic compounds(18)**												
Trimethylamine	20.28(423)	Amine, Fishy	9.58±0.58	ND	2.71±0.34	0.80±0.07	ND	ND	ND	1.46±0.23	3.57±0.23	ND
Diethyl ether	23.72(477)	Etheral	2.87±0.48	ND	ND	1.40±0.35	ND	ND	ND	2.59±0.30	ND	ND
Acetonitrile	26.04(514)	Sweet	ND	ND	ND	ND	ND	8.97±1.71	2.47±0.18	5.28±0.33	0.03±0.06	5.40±0.51

tert-Butyl methyl ether	28.15(548)	Fruity, Ethanol	ND	2.94±0.04	1.30±0.21	ND	ND	ND	ND	ND	3.94±0.49	ND
Diisopropyl ether	33.01(613)	Etheral	ND	ND	ND	ND	ND	ND	ND	ND	0.11±0.02	ND
1-Butanamine	36.61(644)	Fishy	ND	ND	ND	ND	ND	0.17±0.05	0.06±0.05	ND	ND	ND
Pyrazine	45.17(718)	Bitter, Nutty	0.19±0.07	ND	0.11±0.03	0.09±0.03	ND	ND	0.13±0.02	0.25±0.08	ND	ND
Pyrrole	47.65(742)	Coffee, Nutty	ND	ND	ND	ND	ND	ND	0.17±0.09	0.29±0.04	ND	ND
Dimethylformamide	50.95(773)	Amine	ND	ND	ND	ND	ND	ND	ND	0.37±0.03	0.23±0.11	0.14±0.07
Dibutyl ether	59.38(866)	-	ND	ND	ND	ND	ND	ND	ND	ND	0.02±0.04	ND
Pentyl-benzene	73.03(1,164)	Oily	ND	ND	ND	ND	ND	ND	ND	0.10±0.01	0.12±0.02	ND
2-Methoxy-3-sec-butylpyrazine	73.19(1,167)	Carrot, Musty	ND	ND	ND	ND	ND	0.13±0.01	ND	ND	ND	ND
Indole	78.71(1,265)	Burnt, Sweet	ND	ND	ND	ND	ND	ND	0.04±0.03	0.02±0.03	ND	ND
Anethole	81.07(1,310)	Oily, Sweet	ND	ND	ND	ND	ND	ND	0.32±0.05	ND	ND	ND
Methyl eugenol	85.71(1,405)	Fresh, Sweet	ND	ND	ND	ND	ND	ND	0.18±0.01	ND	ND	0.18±0.02
Myristicin	91.25(1,528)	Balsamic	ND	ND	ND	ND	ND	0.61±0.02	0.61±0.06	0.67±0.11	ND	0.57±0.03
Propazine	101.45(1,760)	-	ND	ND	ND	ND	ND	ND	ND	ND	ND	0.11±0.02
Ambroxide	104.69(1,833)	Dry, Sweet	2.02±0.08	ND	1.77±0.12	1.92±0.21	ND	1.90±0.09	1.94±0.15	1.83±0.07	1.95±0.11	1.87±0.12
**Ketones(11)**												
Propan-2-one	24.82(495)	Fruity, Sweet	1.05±0.47	ND	ND	ND	ND	ND	ND	ND	ND	ND
2-Butanone	31.29(600)	Butter, Sweet	ND	3.21±0.08	4.21±0.80	1.46±0.16	0.38±0.01	ND	ND	1.49±0.08	ND	ND
1-Hydroxy-2-propanone	37.11(648)	Sweet	ND	0.22±0.01	ND	ND	ND	ND	0.20±0.05	ND	0.45±0.01	0.21±0.03
2-Pentanone	40.37(675)	Fruity, Sweet	0.47±0.07	ND	ND	ND	0.09±0.02	ND	ND	0.18±0.08	0.22±0.04	ND

3-Penten-2-one	45.11(717)	-	ND	ND	ND	ND	ND	ND	ND	ND	ND	0.10±0.13
2-Hexanone	53.03(793)	Acetone, Fruity	ND	ND	ND	0.04±0.04	1.06±0.62	ND	ND	ND	ND	ND
3-Hexen-2-one	57.93(849)	-	ND	ND	ND	ND	ND	ND	0.21±0.02	ND	0.33±0.01	ND
δ-Hexalactone	68.59(1,085)	Coconut, Creamy	ND	ND	ND	ND	ND	ND	ND	ND	ND	ND
3-Nonen-2-one	71.59(1,140)	Fruity	ND	ND	ND	ND	ND	0.08±0.00	0.07±0.00	ND	0.09±0.01	0.06±0.01
2-Decanone	75.28(1,201)	Citrus	0.06±0.06	ND	ND	ND	0.26±0.09	ND	ND	ND	ND	ND
δ-Octalactone	79.91(1,288)	Coconut, Sweet	ND	ND	ND	ND	ND	ND	ND	0.29±0.07	ND	ND
**Furans(5)**												
Furan	24.71(493)	Etheral	ND	ND	ND	0.27±0.18	ND	ND	ND	ND	ND	0.08±0.13
3-Methylfuran	33.06(614)	-	0.48±0.08	0.14±0.02	ND	ND	ND	ND	ND	ND	ND	ND
2-Ethylfuran	42.45(693)	Burnt, Sweet	ND	8.48±0.46	0.23±0.04	ND	ND	0.06±0.05	ND	2.98±0.33	ND	2.10±0.13
Furfural	55.82(824)	Almond, Sweet	0.15±0.01	0.15±0.01	ND	ND	ND	ND	ND	ND	ND	ND
2-Butylfuran	62.02(898)	Sweet	1.32±0.19	ND	ND	ND	ND	ND	ND	ND	ND	ND
**Sulfur-containing compounds(5)**												
Methanethiol	23.77(478)	Fishy	ND	ND	0.08	ND	0.75	ND	ND	ND	ND	ND
Thiophene	40.41(676)	Sulfurous	ND	ND	0.31±0.27	6.80±0.55	ND	0.77±0.09	ND	2.03±0.33	ND	1.60±0.18
Butanethiol	45.13(717)	Sulfurous	ND	ND	ND	ND	ND	ND	0.19	ND	ND	ND
2-Methylthiophene	50.84(772)	Sulfurous	ND	0.45	ND	0.49	ND	ND	ND	ND	ND	ND

Methional	62.03(898)	Vegetable	ND	ND	ND	ND	ND	ND	1.42	ND	1.11	ND

ASC:Asian_salmon and chicken, AS: Asian_salmon, AP: Asian_poultry, AF: Asian_fish, AC: Asian_chicken, IS:International_salmon, ITS:International_turkey and salmon, IS2:International_salmon, IS3:International_salmon, IS4: :International_salmon. Data represent the mean ± SD in triplicate. 1)RT: retention time 2)RI: retention indices 3)ND: not detected

Supplementary Table S2 Volatile compounds of 10 dog foods using GC/MS

(ug/ g)

**Volatile compounds**	**RT[1] (min)**	**RI[2]**	**I.D.[3]**	**ASC**	**AS**	**AP**	**AF**	**AC**	**IS**	**ITS**	**IS2**	**IS3**	**IS4**
**Acids and esters(29)**													
Ethyl 3-hydroxydodecanoate	6.79	<800	MS	ND[4]	ND	ND	ND	ND	0.06±0.01	ND	ND	ND	ND
2-Ethylhexyl acrylate	7.02	<800	MS	ND	ND	0.19±0.01	ND	ND	ND	ND	ND	ND	ND
Heptanoic acid, 1-methylethyl ester	7.55	<800	MS	ND	ND	ND	ND	ND	0.84±0.01	ND	ND	ND	ND
Hexanoic acid	7.65	<800	MS	0.41±0.01	ND	ND	ND	ND	ND	0.31±0.01	0.25±0.01	ND	ND
Butanoic acid	8.14	815	MS	2.32±0.01	0.57±0.01	ND	0.62±0.19	ND	1.18±0.20	1.17±0.36	1.34±0.76	0.90±0.57	0.97±0.88
3-Methylbutanoic acid	9.59	860	MS	ND	0.22±0.01	ND	0.93±0.36	ND	0.25±0.01	0.36±0.02	ND	0.80±0.37	0.21±0.13
Isovaleric acid	9.77	865	MS	ND	ND	ND	ND	ND	ND	ND	ND	ND	0.51±0.01
2-Methyl octanoic acid	10.07	873	MS	ND	0.29±0.18	ND	ND	ND	ND	0.52±0.01	ND	ND	1.52±0.01
2-Methyl butanoic acid	10.09	874	MS	ND	0.26±0.01	0.31±0.09	ND	ND	0.85±0.01	ND	ND	1.30±0.31	0.60±0.01
Pentanoic acid	11.01	911	MS/RI	ND	0.55±0.01	ND	2.15±1.45	ND	ND	1.01±0.19	1.12±1.01	ND	ND

Propyl-propanedioic acid	11.11	901	MS	ND	ND	ND	ND	ND	1.03±0.01	ND	ND	ND	ND
2-Amino-4-methylbenzoic acid	11.41	911	MS	ND	ND	ND	ND	ND	ND	ND	ND	0.41±0.01	1.08±0.01
4-Methyl-pentanoic acid	12.87	956	MS	0.09±0.01	ND	0.29±0.01	ND	ND	ND	ND	ND	ND	0.175±0.01
Octyl chloroformate	13.53	975	MS	ND	ND	1.07±0.01	ND	ND	ND	ND	ND	ND	ND
Heptanoic acid	14.08	991	MS	1.14±0.01	0.32±0.01	0.28±0.01	0.26±0.01	ND	1.51±1.15	ND	0.83±0.18	ND	0.17±0.01
Sobutyl octyl carbonate	15.26	1028	MS	ND	ND	ND	ND	ND	ND	ND	1.66±0.01	ND	ND
Oxalic acid, 2-ethylhexyl isobutyl ester	15.63	1040	MS	ND	ND	3.46±0.01	ND	ND	ND	ND	ND	ND	ND
Decan-2-yl 2-methylbutanoate	16.2	1058	MS	ND	ND	ND	ND	ND	0.30±0.01	ND	ND	ND	ND
2,4-Hexadienedioic acid	16.81	1077	MS	ND	ND	1.17±0.92	ND	ND	ND	ND	ND	ND	ND
Sorbic acid	16.86	1079	MS	ND	ND	1.36±0.01	ND	ND	ND	ND	ND	ND	ND
Heptanoic acid, ethyl ester	17.55	1099	MS	0.33±0.05	ND	ND	ND	ND	ND	ND	ND	ND	ND
Pentadecyl chloroacetate	17.84	1110	MS	ND	0.25±0.01	ND	ND	ND	ND	ND	ND	ND	ND
Octanoic acid	19.69	1173	MS	0.22±0.01	ND	ND	ND	ND	ND	0.15±0.01	0.27±0.01	ND	ND
Octanoic acid, ethyl ester	20.41	1197	MS	1.17±0.01	0.58±0.29	ND	ND	0.13±0.01	ND	ND	ND	ND	ND

Hexyl-2-methylbutyrate	21.52	1238	MS	ND	ND	ND	ND	ND	ND	0.07±0.01	ND	ND	ND
Hexanedioic acid, dimethyl ester	21.73	1246	MS	ND	ND	ND	ND	ND	ND	ND	0.26±0.01	ND	ND
Nonanoic acid	22.3	1266	MS	ND	ND	ND	ND	ND	ND	0.08±0.01	ND	ND	ND
Decanoic acid, ethyl ester	25.68	1393	MS	ND	1.04±0.59	0.09±0.01	0.12±0.02	0.29±0.01	ND	ND	0.11±0.01	ND	ND
Sulfurous acid, 2-propyl tridecyl ester	25.77	1396	MS	ND	ND	0.07±0.01	ND	ND	ND	ND	ND	ND	ND
**Alcohols(19)**													
Isoamyl alcohol	6.09	<800	MS	1.00±0.01	ND	ND	ND	ND	ND	ND	ND	ND	ND
1-Pentanol	6.12	<800	MS	0.81±0.64	ND	0.83±0.07	ND	ND	ND	ND	ND	ND	ND
1-Butoxy-2-propanol	12.61	949	MS	ND	ND	ND	ND	ND	ND	ND	ND	0.65±0.05	ND
Heptanol	13.52	975	MS	ND	ND	1.17±0.01	ND	ND	ND	ND	ND	ND	ND
1-Octen-3-ol	13.84	984	MS	ND	5.06±0.01	3.69±0.01	ND	ND	ND	ND	ND	ND	ND
1-Ethynylcyclopentanol	15.52	1037	MS	ND	0.72±0.01	ND	ND	ND	ND	ND	ND	ND	ND
3,5-Octadien-2-ol	15.74	1044	MS	0.50±0.01	1.23±0.01	ND	ND	ND	ND	ND	ND	ND	ND
Cyclohexanol	16.21	1059	MS	0.69±0.01	ND	ND	ND	ND	ND	ND	ND	ND	ND
1-Octanol	16.7	1074	MS	ND	0.75±0.01	ND	ND	ND	ND	ND	ND	ND	ND
4-Cresol	16.82	1077	MS	ND	ND	ND	ND	ND	0.34±0.05	ND	ND	ND	ND
9-Octadecen-1-ol	18.72	1141	MS	ND	ND	ND	ND	0.05±0.01	ND	ND	ND	ND	ND

4-Terpineol	19.94	1181	MS	ND	ND	ND	ND	ND	ND	0.23±0.01	ND	ND	ND
1-Dodecanethiol	20.25	1191	MS	ND	ND	ND	ND	ND	ND	0.19±0.01	ND	ND	ND
1-Tetradecanol	20.26	1192	MS	ND	ND	ND	ND	ND	ND	ND	0.04±0.01	ND	ND
2-Hexyl-1-octanol	23.62	1314	MS	0.07±0.01	ND	ND	ND	ND	ND	ND	ND	ND	ND
2-Vinylguaiacol	23.67	1316	MS	ND	ND	ND	ND	ND	ND	ND	0.23±0.16	ND	ND
2-Butyl-2,7-octadien-1-ol	25.17	1374	MS	ND	0.11±0.09	0.08±0.01	ND	ND	ND	ND	ND	ND	ND
4-Benzene-1,2-diol	25.93	1403	MS	0.04±0.01	ND	ND	ND	ND	ND	ND	ND	ND	ND
p-Guaiacol	27.97	1486	MS	0.03±0.01	ND	ND	ND	ND	ND	ND	ND	ND	ND
2,6-bis-*p*-Cresol	27.59	1471	MS	0.03±0.01	0.29±0.18	0.18±0.03	ND	ND	0.11±0.02	0.18±0.01	ND	ND	ND
**Aldehydes(17)**													
3-Methyl-butanal	4.41	<800	MS	0.03±0.01	0.75±0.01	0.04±0.01	ND	0.29±0.01	ND	ND	ND	ND	ND
Pentanal	4.61	<800	MS	ND	0.26±0.01	ND	ND	ND	ND	ND	ND	ND	ND
2-Pentenal	6.57	<800	MS	ND	ND	ND	ND	ND	ND	ND	ND	ND	0.07±0.01
3-Methoxy-propanal	7.72	<800	MS	ND	ND	ND	ND	ND	ND	ND	0.22±0.01	ND	ND
Hexanal	7.95	808	MS/RI	ND	18.26±16.62	46.21±1.76	3.42±0.01	4.60±0.94	ND	ND	ND	ND	4.71±1.61
3-Furaldehyde	9.02	843	MS	ND	ND	ND	ND	ND	ND	ND	0.09±0.01	ND	ND
4-Hexenal	9.78	865	MS	2.73±0.01	ND	ND	ND	ND	ND	ND	ND	ND	ND
Heptanal	11.27	906	MS/RI	ND	1.61±0.01	ND	ND	ND	0.77±0.01	ND	ND	0.66±0.01	0.85±0.60
Benzaldehyde	13.22	966	MS/RI	2.15±0.01	2.59±1.83	2.35±0.08	1.52±0.33	0.71±0.37	ND	ND	2.86±1.06	2.66±0.42	1.85±0.55

Octanal	14.59	1005	MS/RI	2.80±0.21	3.26±1.02	3.64±1.19	ND	ND	ND	ND	ND	ND	ND
2,4-Heptadienal	14.83	1014	MS	ND	ND	ND	ND	1.87±0.04	ND	ND	ND	ND	ND
Benzeneacetaldehyde	15.9	1049	MS	ND	0.78±0.01	ND	ND	ND	0.35±0.01	ND	0.89±0.01	ND	ND
Nonanal	17.72	1105	MS/RI	1.98±0.28	2.47±1.11	3.65±1.13	1.00±0.20	1.25±0.01	0.73±0.18	0.80±0.02	1.44±0.49	0.71±0.24	9.49±11.61
2,5-bis-benzaldehyde	17.99	1115	MS	ND	ND	ND	ND	ND	ND	ND	ND	ND	1.00±0.01
Decanal	20.67	1206	MS/RI	0.07±0.01	ND	0.10±0.01	ND	ND	ND	ND	ND	ND	ND
β-Cyclocitral	21.17	1225	MS	0.07±0.01	ND	ND	ND	ND	0.03±0.01	0.07±0.01	ND	ND	ND
2-Butyl-2-octenal	25.17	1374	MS	ND	ND	0.07±0.01	ND	ND	ND	ND	0.09±0.01	ND	ND
**Hydrocabons(67)**													
Methylbenzene	6.99	<800	MS	ND	12.44±6.65	ND	2.85±0.01	ND	ND	0.71±0.23	ND	ND	2.12±0.06
1,3,5-Cycloheptatriene	7.15	<800	MS	ND	ND	ND	ND	ND	ND	0.51±0.01	0.19±0.01	ND	ND
Ethylbenzene	9.93	870	MS	ND	ND	ND	ND	0.14±0.01	ND	ND	ND	ND	ND
4-Methyl-octane	9.99	871	MS	1.95±0.83	ND	ND	ND	ND	ND	ND	0.22±0.01	ND	ND
1,4-Dimethylbenzene	10.18	876	MS	ND	ND	ND	2.00±1.07	ND	ND	ND	ND	ND	ND
1,3-Dimethylbenzene	10.26	879	MS	ND	1.47±0.01	ND	1.48±0.01	ND	ND	ND	ND	ND	ND
Pulegone	11.64	918	MS	ND	ND	0.76±0.01	ND	ND	ND	ND	ND	ND	ND
Sulfonylbis-methane	11.81	924	MS	ND	ND	ND	ND	0.83±0.01	ND	ND	ND	ND	ND

trans-Ocimene	12.34	940	MS	ND	ND	ND	ND	0.03±0.01	ND	ND	ND	ND	ND
α-Pinene	12.33	941	MS	ND	ND	ND	ND	ND	0.40±0.09	0.35±0.19	ND	0.57±0.06	0.37±0.01
1,2,3-Trimethylcyclopentane	13.54	976	MS	ND	2.42±0.92	ND	ND	ND	ND	ND	ND	ND	ND
2-Decene	13.65	979	MS	ND	ND	ND	0.46±0.01	ND	ND	ND	ND	ND	ND
Fucose	14.01	989	MS	ND	ND	0.76±0.01	ND	ND	ND	ND	ND	ND	ND
2,2,4,6,6-Pentamethylheptan	14.18	993	MS	ND	ND	ND	ND	ND	ND	ND	ND	4.60±0.01	5.83±0.01
2,2-Dimethyltetradecane	15.23	1027	MS	ND	ND	ND	ND	ND	ND	1.40±0.07	ND	ND	1.96±0.69
1-Ethyl-2,4-dimethylbenzene	15.31	1030	MS	ND	0.35±0.01	ND	ND	ND	ND	ND	ND	ND	ND
3-Methyloctane	15.38	1032	MS	ND	ND	ND	0.93±0.01	ND	ND	ND	ND	ND	ND
2,5-Dimethylheptane	15.4	1033	MS	ND	ND	1.53±0.01	ND	ND	ND	ND	ND	ND	ND
Limonene	15.44	1034	MS/RI	ND	ND	ND	ND	ND	0.53±0.13	ND	ND	ND	ND
2,2,8-Trimethyldecane	15.47	1035	MS	ND	ND	ND	ND	ND	ND	ND	1.38±0.01	ND	ND
3-Ethyl-2-methyl-1,3-hexadiene	15.5	1036	MS	0.69±0.01	1.10±0.01	0.75±0.01	ND	ND	ND	ND	ND	ND	ND
3,3-Dimethylhexane	15.56	1038	MS	ND	ND	ND	ND	ND	ND	ND	ND	ND	3.48±0.01
2,6,10-Trimethyldodecane	15.63	1040	MS	ND	ND	2.90±0.01	ND	ND	ND	ND	ND	ND	ND

5-Methyl-octadecane,	15.9	1049	MS	ND	ND	ND	0.52±0.16	ND	ND	ND	ND	ND	ND
Propyl-cyclopentane	15.97	1051	MS	ND	ND	ND	0.06±0.01	ND	ND	ND	ND	ND	ND
6-Methyl-tridecane	16.03	1053	MS	ND	ND	ND	ND	ND	ND	ND	ND	ND	0.24±0.01
2,6,8-Trimethyl-decane	16.06	1054	MS	ND	ND	1.72±0.01	ND	ND	ND	ND	ND	ND	ND
5-Butylnonane	16.09	1055	MS	0.49±0.01	ND	ND	ND	ND	ND	ND	ND	ND	ND
7,9-Dimethylhexadecane	16.1	1055	MS	ND	ND	ND	ND	ND	ND	ND	0.52±0.01	ND	ND
2-Methyldecane	16.19	1058	MS	ND	ND	ND	2.08±0.15	ND	ND	1.45±0.01	1.94±0.01	ND	1.90±0.01
2,3-Epoxyheptane	16.21	1059	MS	0.68±0.01	ND	ND	ND	ND	ND	ND	ND	ND	ND
2,2,3,4-Tetramethylpentane	16.27	1061	MS	ND	ND	0.58±0.01	ND	ND	ND	ND	ND	ND	ND
2,3,6-Trimethyldecane	16.32	1062	MS	ND	ND	ND	ND	ND	ND	ND	3.56±0.01	ND	ND
3-Methylhexadecane	16.48	1067	MS	ND	ND	0.49±0.01	ND	ND	ND	ND	ND	ND	ND
3,8-Dimethyldecane	16.49	1068	MS	ND	ND	ND	ND	ND	ND	ND	2.96±0.01	ND	ND
5-Methyltridecane	16.69	1074	MS	ND	1.41±0.01	ND	1.68±0.01	ND	ND	ND	ND	ND	ND
4,8-Dimethylundecane	16.85	1078	MS	ND	ND	ND	1.86±0.01	ND	ND	ND	ND	ND	ND

2,8-Dimethyl-undecane	16.87	1079	MS	ND	ND	ND	ND	ND	ND	1.44±0.03	ND	ND	2.88±1.49
7-Methylhexadecane	17.04	1084	MS	ND	ND	ND	0.56±0.01	ND	ND	ND	ND	ND	ND
2,3,6,7-Tetramethyloctane	17.11	1086	MS	ND	ND	ND	ND	0.90±0.01	ND	ND	2.00±0.01	2.08±0.01	ND
3-Ethyltetracosane	17.17	1088	MS	ND	ND	ND	ND	ND	ND	ND	ND	ND	0.49±0.01
3,3-Dimethylhexane	17.27	1091	MS	ND	ND	ND	ND	0.47±0.01	ND	ND	1.53±0.01	ND	ND
2-Methyldodecane	17.56	1099	MS	ND	ND	ND	0.32±0.01	ND	ND	ND	ND	ND	ND
4-Methylundecane	17.67	1103	MS	ND	ND	ND	ND	ND	ND	ND	4.46±0.90	ND	ND
Dimethyl-4-aniline	18	1115	MS	ND	ND	ND	ND	ND	ND	ND	ND	2.81±0.01	ND
1-Ethyl-3,5-dimethylbenzene	18.24	1124	MS	ND	ND	ND	0.19±0.08	ND	ND	ND	ND	ND	ND
2,2,6-Trimethyldecane	18.32	1127	MS	ND	ND	ND	ND	ND	ND	ND	0.47±0.01	ND	ND
Tridecane	18.41	1129	MS	ND	ND	ND	ND	0.30±0.01	ND	ND	ND	0.16±0.01	ND
2,3,5-Trimethyldecane	18.88	1146	MS	ND	ND	ND	ND	ND	ND	ND	ND	ND	0.19±0.01
6,6-Diethylhoctadecane	18.9	1147	MS	ND	0.03±0.01	ND	ND	ND	ND	ND	ND	ND	ND

2-Butyl-1-decene	19.55	1169	MS	ND	ND	ND	ND	ND	ND	ND	ND	ND	0.14±0.01
3-Methyl-undecane	19.65	1172	MS	ND	0.52±0.01	ND	ND	ND	ND	ND	ND	ND	ND
1-Tridecene	20.25	1130	MS	ND	ND	ND	ND	ND	ND	0.20±0.01	ND	ND	0.29±0.01
Dodecane	20.47	1198	MS/RI	ND	0.49±0.23	0.49±0.04	0.12±0.03	0.45±0.01	ND	ND	ND	0.28±0.10	0.77±0.45
8-Methyl-heptadecane	22.7	1280	MS	1.76±0.01	ND	ND	ND	ND	ND	ND	ND	ND	ND
1-Methyl-4-cyclohexane	23.13	1295	MS	ND	ND	ND	ND	ND	ND	0.56±0.01	ND	ND	ND
2,4-Dimethyldecane	23.21	1298	MS	ND	ND	ND	ND	ND	ND	ND	0.04±0.01	ND	ND
2-Methyldodecane	23.28	1300	MS	0.34±0.01	ND	ND	ND	ND	ND	ND	ND	ND	ND
3,3-Dimethylhexane	24.15	1335	MS	0.59±0.01	ND	ND	ND	ND	ND	ND	ND	ND	ND
2-Isopropenyl-*p*-cymene	24.85	1362	MS	0.04±0.01	ND	ND	ND	ND	ND	ND	ND	ND	ND
3-Methylheneicosane	25.04	1369	MS	ND	0.04±0.01	ND	ND	ND	ND	ND	ND	ND	ND
Tetradecane	25.77	1397	MS/RI	ND	ND	ND	ND	0.08±0.01	ND	ND	0.13±0.01	ND	0.16±0.01
trans-Caryophyllene	26.49	1426	MS	ND	ND	ND	ND	ND	ND	0.09±0.01	ND	ND	ND
Tritetracontane	28.02	1488	MS	0.05±0.01	ND	ND	ND	ND	ND	ND	ND	ND	ND
2,6,10-Trimethylpentadecane	29.09	1533	MS	ND	ND	ND	ND	ND	ND	ND	0.03±0.01	ND	ND

Hentriacontane	29.18	1537	MS	0.14±0.01	ND	ND	ND	ND	ND	ND	ND	ND	ND
Tetracosane	29.41	1547	MS	ND	ND	ND	ND	ND	ND	ND	0.06±0.01	ND	ND
**Heterocyclic compounds(21)**													
Trimethylamine	2.16	<800	MS	ND	ND	ND	ND	ND	ND	ND	ND	0.46±0.01	ND
1,2-Dimethylhydrazine	3.72	<800	MS	ND	ND	ND	ND	ND	ND	ND	0.23±0.15	ND	ND
Ethyl-1,3-dithioisoindoline	8.45	825	MS	ND	0.38±0.01	ND	ND	ND	4.87±0.01	ND	ND	ND	ND
3,5-Dimethylpyrazole	9.1	845	MS	1.74±0.01	ND	ND	ND	ND	ND	ND	ND	ND	ND
Methoxy-phenyl-oxime	11.34	909	MS	ND	ND	ND	ND	4.07±0.01	ND	ND	ND	ND	ND
2,5-Dimethylpyrazine	11.57	916	MS	ND	ND	ND	ND	ND	0.74±0.01	ND	ND	ND	ND
2,6-Dimethylpyrazine	11.6	917	MS	0.70±0.01	1.27±0.84	0.57±0.01	ND	ND	0.17±0.01	ND	ND	ND	ND
1-Nonyne	15.23	1027	MS	ND	ND	ND	ND	ND	0.34±0.01	ND	ND	ND	ND
Phytane	15.72	1043	MS	ND	ND	ND	0.89±00.01	ND	ND	ND	ND	ND	2.09±0.01
2-Acetylpyrrole	16.4	1065	MS	ND	ND	ND	ND	ND	0.09±0.01	ND	ND	0.37±0.10	ND
2,6-Diethylpyrazine	16.95	1081	MS	ND	ND	ND	ND	ND	ND	ND	ND	0.91±0.14	ND

2-Ethyl-3,5-dimethyl-pyrazine	16.97	1082	MS	1.16±0.08	0.89±0.28	1.03±0.01	1.22±0.31	0.65±0.01	0.49±0.08	ND	ND	ND	ND
Tetramethyl-pyrazine	17.21	1089	MS	ND	1.38±0.44	ND	ND	ND	ND	ND	ND	ND	ND
3-Methyl-1H-indazole	17.58	1100	MS	ND	ND	ND	ND	ND	0.16±0.01	ND	ND	ND	ND
2-Droxybenzylpyrrole	17.9	1112	MS	2.83±0.01	ND	1.31±0.01	ND	ND	ND	ND	0.67±0.01	ND	ND
Maltol	17.94	1113	MS	ND	ND	ND	ND	ND	ND	ND	ND	0.35±0.01	ND
2-3-Indole	19	1151	MS	0.14±0.01	0.25±0.01	ND	ND	ND	0.23±0.01	ND	0.39±0.01	0.26±0.01	ND
Hexadecylene oxide	20.66	1205	MS	ND	0.06±0.01	ND	ND	ND	ND	ND	ND	ND	ND
1-2-Pyrrolidon	20.86	1213	MS	ND	ND	ND	ND	ND	ND	ND	ND	ND	0.12±0.01
Butylhydroxyanisole	27.96	1486	MS	ND	0.07±0.05	ND	0.42±0.01	ND	ND	ND	0.72±0.58	ND	0.11±0.01
Dihydroactinidiolide	29.13	1535	MS	ND	ND	ND	ND	ND	0.04±0.01	ND	ND	ND	ND
**Ketones(18)**													
2-Heptanone	10.95	897	MS	ND	ND	0.03±0.03	ND	0.80±0.32	ND	ND	ND	ND	ND
Dimethyl sulfone	11.83	924	MS/RI	ND	ND	ND	ND	0.73±0.01	ND	ND	ND	ND	ND
4,4-Dimethylcyclohexan-1-one	13.54	976	MS	0.86±0.01	ND	ND	ND	ND	ND	ND	ND	ND	ND
2,3-Octanedione	14.01	988	MS	ND	1.81±0.01	ND	ND	ND	ND	ND	ND	ND	ND
6-Methyl-5-hepten-2-one	14.06	990	MS	ND	ND	ND	ND	ND	ND	ND	ND	ND	2.03±0.01
3-Octen-2-one	15.72	1043	MS/RI	0.54±0.01	0.80±0.01	1.24±0.01	ND	ND	ND	ND	ND	ND	ND

γ-Hexalactone	16.22	1059	MS	ND	0.82±0.22	ND	ND	ND	ND	ND	ND	ND	ND
1-Phenylethanone	16.6	1071	MS	ND	ND	ND	ND	0.62±0.01	ND	ND	ND	ND	ND
3,5-Octadiene-2-one	16.71	1074	MS	ND	ND	ND	ND	5.83±2.17	0.86±0.21	3.14±0.12	ND	ND	ND
2-Nonanone	17.36	1094	MS	0.62±0.05	1.22±0.77	ND	ND	ND	ND	ND	ND	ND	ND
5-Methyl-2-cyclohexanone	19.57	1169	MS	ND	ND	ND	ND	ND	ND	2.08±0.01	ND	ND	ND
2-Piperidinone	19.81	1177	MS	ND	0.24±0.01	ND	0.17±0.07	ND	ND	ND	ND	1.46±0.44	0.29±0.20
2-Decanone	20.3	1193	MS	0.34±0.08	ND	ND	0.10±0.01	ND	ND	ND	0.25±0.15	ND	ND
tert-Butyl-*p*-benzoquinone	20.6	1203	MS	ND	ND	ND	ND	ND	0.84±0.01	ND	ND	ND	ND
Chrysanthenone	20.88	1214	MS	ND	0.30±0.01	ND	ND	ND	ND	ND	ND	ND	ND
Carvone	21.8	1248	MS	ND	ND	ND	ND	ND	ND	0.28±0.01	ND	ND	ND
3-Butyl-2-cyclohexen-1-one	22.41	1270	MS	0.07±0.01	ND	ND	ND	ND	ND	ND	ND	ND	ND
2,5-Dimethyl-crotonophenone	24.83	1361	MS	ND	ND	0.03±0.01	ND	ND	ND	ND	ND	ND	ND
**Furans(7)**													
2,5-Dimethylfuran	9.06	844	MS	1.18±0.01	ND	ND	ND	ND	ND	ND	0.92±0.01	ND	ND
2-Furancarboxaldehyde	9.08	845	MS	0.97±0.01	ND	ND	0.44±0.01	ND	ND	ND	0.31±0.08	ND	ND
Furfural	9.15	847	MS	1.30±0.01	ND	ND	ND	ND	ND	ND	0.20±0.01	ND	ND

2-Furanmethanol	9.76	865	MS	5.18±0.23	ND	ND	ND	ND	ND	ND	ND	ND	ND
2-Pentylfuran	14.22	994	MS	8.50±0.70	13.14±3.29	14.37±0.25	3.97±0.58	6.54±0.62	1.55±0.32	5.92±0.25	8.29±2.02	ND	ND
3-Methylbenzofuran	17.58	1100	MS	ND	ND	ND	ND	ND	0.25±0.01	ND	ND	ND	ND
5-Heptyldihydro-2-furanone	24.92	1365	MS	0.07±0.01	ND	ND	ND	ND	ND	ND	ND	ND	ND
**Sulfur-containing compounds(2)**													
1-Dodecanethiol	20.25	1191	MS	ND	ND	ND	ND	ND	ND	0.19±0.01	ND	ND	ND
2-Formyl-3-methylthiophene	18.27	1125	MS	ND	ND	ND	ND	ND	ND	ND	ND	0.41±0.01	ND

ASC: Asian_salmon and chicken, AS: Asian_salmon, AP: Asian_poultry, AF: Asian_fish, AC: Asian_chicken, IS: International_salmon, IST: International_turkey and salmon, IS2: International_salmon, IS3: International_salmon, IS4: International_salmon. Data represent the mean ± SD in triplicate.
1)RT: retention time 2)RI: retention indices 3)I.D.: identification 4)ND: not detected
